# Cholera in the Era of COVID-19 Pandemic: A Worrying Trend in Africa?

**DOI:** 10.3389/ijph.2021.1604030

**Published:** 2021-06-15

**Authors:** Oloche Owoicho, Priscilla Abechi, Charles Ochieng’ Olwal

**Affiliations:** ^1^ West African Centre for Cell Biology of Infectious Pathogens (WACCBIP), University of Ghana, Accra, Ghana; ^2^ Department of Biological Sciences, Benue State University, Makurdi, Nigeria; ^3^ Department of Biochemistry, Cell and Molecular Biology, College of Basic and Applied Sciences, University of Ghana, Accra, Ghana; ^4^ African Centre of Excellence for Genomics of Infectious Diseases, Redeemer’s University, Ede, Nigeria

**Keywords:** COVID-19 pandemic, SARS-CoV-2, cholera, *Vibrio cholerae*, WASH

**The IJPH series “Young Researcher Editorial” is a training project of the Swiss School of Public Health**.

Since 2019 the Coronavirus Disease 2019 (COVID-2019) pandemic has caused a massive increase in mortality and strained health care facilities around the world. In Africa COVID-19 has caused more than 100,000 associated deaths [1] and slowed the fight against deadly infectious diseases like malaria, tuberculosis, HIV/AIDS and neglected tropical diseases [2, 3]. It may also have slowed progress against cholera.

Amid the COVID-19 pandemic, Burundi, Benin, Cameroon, Democratic Republic of Congo, Ethiopia, Kenya, Mozambique, Nigeria, Togo and Uganda have reported cholera outbreaks [4, 5]. In non-pandemic years, there are about 2.9 million cholera cases and 95,000 associated annual deaths in 69 cholera-endemic countries, most of which are within the sub-Saharan Africa [7]. Cholera is an acute watery diarrhea caused by toxigenic strains of *Vibrio cholerae*, particularly serogroups O1 and O139, characterized by excessive dehydration and a high fatality rate [6]. It is acquired fecal-orally via contaminated food and water. Contaminated drinking water, poor sanitation and hygiene, fragile health care systems, humanitarian crises and hydroclimatic factors account for the disproportionate burden of cholera in sub-Saharan Africa [8, 9].

In 2019, 16 African countries reported 55,087 cholera cases, with a case fatality rate (CFR) of 1.6%—a considerable decrease from the 2.0% reported in 2018 [10]. Cholera CFR increased in 2020; for example, in Central and West Africa, CFR rose from 1.8% in 2019 to 2.1% in 2020 [11]. But the number of cholera cases decreased from 34,957 to 23,628 in the same two sub-regions [11]. At the country level, from 2019 to 2020, cholera CFR rose by 2.2% in Benin, 0.4% in Cameroon, 1.2% in Liberia, and 3.5% in Nigeria [11]. In Nigeria, the 2020 outbreak extended into 2021, comprising 266 suspected cases from geographically distinct communities, and reported a CFR of 12.0% from December 2020 to January 2021 [12]. Collectively, these statistics show that meeting the Global Task Force on Cholera Control’s 2030 target of reducing cholera deaths by 90% will require stepping up the fight against cholera in sub-Saharan Africa, even amid the pandemic.

The pandemic may have a negative effect on key cholera control components like surveillance, a key measure against cholera outbreak and associated mortality. Effective surveillance is needed to detect *V. cholerae* early in clinical, environmental, or food samples. When health workers and funds are reassigned to fight the COVID-19 pandemic, fewer health workers and less funding may be available for cholera surveillance, especially in resource-limited settings like sub-Saharan Africa. Since CFR is a function of disease cases, the increased cholera CFR in 2020 in Central and West Africa may have resulted from reduced surveillance, more severe cholera, and the higher number of cholera-related deaths.

COVID-19 may also have decreased health promotion activities that mitigate cholera outbreaks. Health promotion complements other cholera control and/or prevention factors like access to potable water, sanitation, and hygiene (WASH). COVID-19 restrictions on transportation and assembly during the pandemic may hinder community-oriented health education and promotion activities that could help control cholera outbreaks. Though the increase in hand washing and/or sanitization in response to the COVID-19 pandemic is expected decrease food- and waterborne infections, cholera statistics from sub-Saharan African countries do not seem to support this hypothesis. We think that limited access to clean water and poor waste disposal in many rural areas and peri-urban slums in sub-Saharan Africa makes it hard to implement hand hygiene effectively.

Finally, COVID-19 may reduce the rate of vaccination against cholera in sub-Saharan Africa. Sustained cholera control in low- and middle-income countries (LMICs) depends on administering oral cholera vaccines but routine vaccinations against infectious diseases, including cholera, were disrupted in many LMICs in the early phase of the COVID-19 pandemic [13].

Although the statistics we cited were mainly gathered from the Central and West African regions, the situation is similar throughout sub-Saharan Africa [14]. While this editorial was under review, Hassan and Nellums [15] provided evidence that supports the view that cholera is a threat in most sub-Saharan African countries. Specifically, they highlighted the threat of cholera outbreak in Ethiopia and Sudan, where ongoing humanitarian crises and COVID-19 compounded the difficulties of stemming the cholera epidemic [15]. There is need for more deliberate efforts to prevent and control cholera in sub-Saharan Africa, during and beyond the pandemic.

To end cholera in sub-Saharan Africa, nations should consider investing in infrastructure like potable water supplies, especially in rural communities and peri-urban slums, since they are hot-spots for cholera outbreaks. Health care systems in sub-Saharan Africa should be strengthened through capacity building to ensure there are enough trained local health care workers. Though the scale of the COVID-19 pandemic exceeds that of most infectious diseases in sub-Saharan Africa, countries where cholera is endemic still need to allocate financial resources and human capital to prevent outbreaks.
